# Reflectance Confocal Microscopy in Monitoring Atopic Dermatitis Treated with Topical Calcineurin Inhibitors

**DOI:** 10.3390/healthcare11020152

**Published:** 2023-01-04

**Authors:** Dalia Bratu, Daniel Boda, Constantin Caruntu

**Affiliations:** 1 Department of Dermatology, ‘Colentina’ Clinical Hospital, 020125 Bucharest, Romania; 2 Department of Dermatology, ‘Carol Davila’ University of Medicine and Pharmacy, 050474 Bucharest, Romania; 3 Department of Dermatology, ‘Ponderas’ Academic Hospital, 014142 Bucharest, Romania; 4 Department of Dermatology, “Prof. N.C. Paulescu” National Institute of Diabetes, Nutrition and Metabolic Diseases, 011233 Bucharest, Romania; 5 Department of Physiology, “Carol Davila” University of Medicine and Pharmacy, 050474 Bucharest, Romania

**Keywords:** atopic dermatitis, reflectance confocal microscopy, virtual biopsy

## Abstract

Atopic dermatitis is a chronic inflammatory skin disease associated with multiple allergies in the atopic march. It has a complex pathogenesis, related to genetic, immune, and environmental factors. Its incidence and prevalence are increasing in the last decades, especially in developed countries. It affects the quality of life due to the recurrent lesions and the associated pruritus. Thus, it is very important to use non-invasive techniques to manage and follow-up the patients with such a heterogenous disease that can have a high impact on some of them. The reflectance confocal microscope is a modern device for in vivo visualization of the epidermis and the upper dermis which could replace in some cases the cutaneous biopsy. We report a case of a patient with atopic dermatitis investigated with the confocal reflectance microscope at the beginning of the topical treatment with calcineurin inhibitors and three weeks after, with favorable evolution. Reflectance confocal microscopy allows the assessment of the dynamic changes in the skin during treatment. Moreover, it can be useful for highlighting discrete changes even in the subclinical stages of the inflammatory process. Future developments, which will lead to the definition and validation of reflectance confocal microscopy criteria for the diagnosis and staging of atopic dermatitis, could help to improve the treatment and prevention strategies of the disease.

## 1. Introduction

Atopic dermatitis (AD), also known as atopic eczema [1], is a chronic but intermittent inflammatory skin disease [1,2,3,4,5]. When symptomatic, it affects the quality of life, with increased physiological stress [6], low self-esteem [1], depression, sleep disturbances, anxiety [7,8,9,10,11], high suicidal risk [1,12,13,14,15,16,17]. It is included in the atopic march, together with alimentary allergies, allergic rhinitis (hay fever), asthma and eosinophilic esophagitis [7,18,19,20,21]. During the last decades, the incidence and prevalence of AD have increased, especially in developed countries [20,22,23,24,25], and are related to genetic, immune, and environmental factors [1,15,16]. The genetic factors increase the susceptibility for atopy but they do not necessarily lead to the expression of it. The atopic phenotype is recessive or polygenic and its expression is the result of the complex interaction between skin barrier dysfunction, immunity dysregulation, and environment [1,15,16]. Chronic exposure, whether intermittent or persistent, to external risk factors such as pollution, toxins, and skin irritants leads to epigenetic changes: DNA methylation, histone modification, and non-coding RNA-dependent mechanisms, with the environment playing an important role in the development of atopic dermatitis [26,27,28,29,30,31,32,33,34,35,36,37,38]. Both females and males are affected [39,40,41], with a peak in occurrence during the first year of life [42].

Clinically, the chronic or relapsing lesions appear in specific areas: face, trunk, extensors, hands as papules or different sizes of plaques, sparing the groin and the axillary region [43]. They can be acute (erythematous papules or vesicles eroded, crusted, oozing), subacute (excoriated papules or plaques) or chronic (lichenified, thick, hyperpigmented) [44]. Children have more widespread lesions involving the head, face, trunk, and arms. Adults typically present with lesions more circumscribed, usually on the arms, legs, hands, neck, and periorbital areas. If the associated pruritus is severe, it can lead to excoriations, especially on the extremities, with bleeding, crusts, lichenification, nodular lesions, hypopigmentation, or hyperpigmentation [39,43,45,46]. Laboratory abnormalities findings include high levels of IgE and high levels of circulating eosinophils, but they are not specific; the intrinsic variant of AD has normal levels [43].

The treatment has to be adapted to the severity of the disease. Those suffering from mild to moderate disease should start with emollients, low-potency topical corticosteroids, and calcineurin inhibitors for special areas. In moderate disease, medium to high potency topical corticosteroids can be used, and high to super high potency topical corticosteroids in short courses [47]. For moderate to severe disease patients may need phototherapy or systemic immunomodulatory therapy with cyclosporine, dupilumab, methotrexate, and azathioprine [47,48,49,50]. Associated bacterial, viral, or fungal infections have to be treated with specific treatments [47].

Reflectance confocal microscopy (RCM) is a non-invasive technique that provides in vivo imaging of the epidermis and the superior dermis [51,52]. It allows the direct evaluation of exocytosis, spongiosis of the spinous and granular layer, parakeratosis, and structural defects of the stratum corneum [51]. It can be very useful in monitoring the dynamic changes of the patient’s skin during the treatment because the images are taken in real time, directly from the skin, unlike skin biopsies [51,53].

Here we report a case of a patient with atopic dermatitis investigated with a reflectance confocal microscope at the beginning of the topical treatment with calcineurin inhibitors and three weeks after, with favorable evolution.

## 2. Case Report

**Figure 1:**
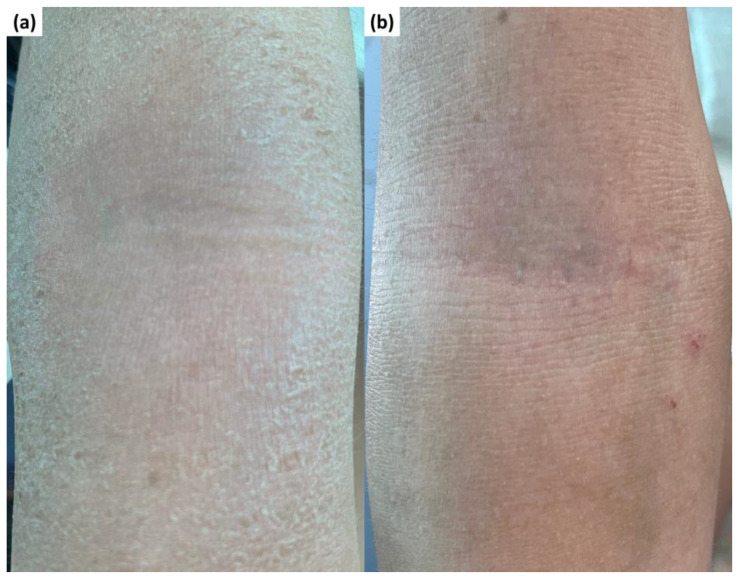
Clinical aspect of the skin (**a**) before treatment—chronic lichenified pruritic plaque associated with scraping injuries localized on the right antecubital fossae; (**b**) 3 weeks after starting treatment—favorable evolution of the lesion.

A 12-year-old female patient with no previously registered medical history was listed at the Department of Dermatology of Ponderas Clinical Hospital for chronic lichenified pruritic plaques associated with scraping injuries localized on the antecubital fossae (Figure 1a). Anamnestically, the lesions occurred intermittently and worsened during the cold season. The patient has no personal history of atopic diseases but her sister is suffering from allergic asthma since the age of 6.

The entire skin evaluation revealed associated eczematous acute plaques on the abdomen, xerosis, keratosis pilaris, cheilitis, palmar hyperlinearity, Dennie–Morgan lines, and Hertoghe’s sign. The recorded serum levels of immunoglobulin E were within the normal range. According to the Hanifin and Rajka criteria [54], the patient has all four major criteria and five of the minor ones associated with atopic dermatitis.

We performed an RCM examination of the skin from the right antecubital fossae before (Figure 1a) and three weeks after starting topical treatment with calcineurin inhibitors (Figure 1b) but also from the right forearm where, from a clinical point of view, the skin appeared healthy.

The patient was treated with topical calcineurin inhibitors on the antecubital fossae because they are an alternative to the topical corticosteroids on the special sites (face, eyelid, neck, folds). These immunomodulating agents do not cause secondary atrophy, a well-known side effect of dermatocorticoids [47,55]. After the initial treatment, the lesions almost healed (Figure 1b), but the maintenance treatment with topical calcineurin inhibitors or dermatocorticoids and emollients after shower are crucial to prevent the recurrence of the lesions or the development of new ones.

**Figure 2:**
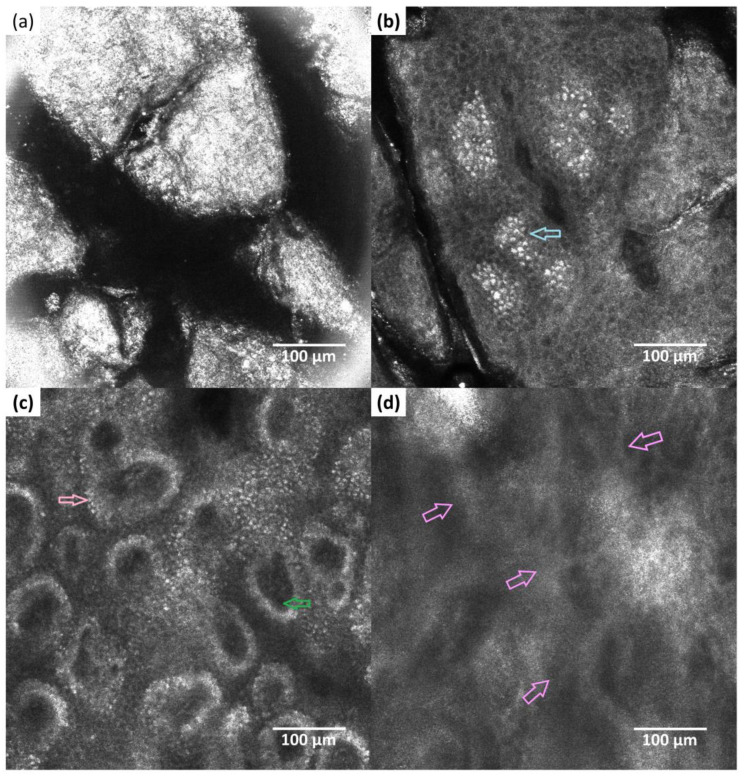
Reflectance confocal microscopy (RCM) images (500 × 500 μm) of the normal skin—right forearm (**a**) cornified layer; (**b**) granular/spinous layer with regular honeycomb pattern; areas with cobblestone pattern (blue arrow) (**c**) dermal-epidermal junction—small basal cells and melanocytes arranged in a circular pattern (bright rings) (pink arrow) surrounding the dermal papillae (dark structures); small blood vessels inside the dermal papillae (green arrow); (**d**) dermis with elastic and collagen fibers visible as bright fibrillar or reticular structures (purple arrow).

**Figure 3:**
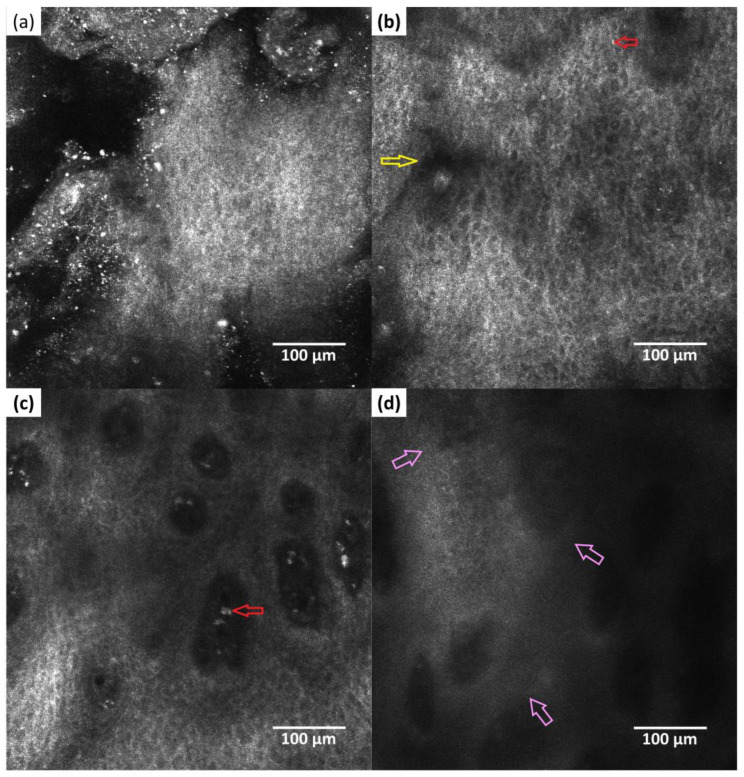
Reflectance confocal microscopy (RCM) images (500 × 500 μm) of the atopic skin—right antecubital fossae (**a**) cornified layer; (**b**) granular/spinous layer with irregular cells, intercellular inflammatory infiltrate (red arrow) and spongiosis (large dark areas) (yellow arrow); (**c**) blurred image of the dermo-epidermal junction with irregular or even absent papillary rings, disappeared cellular borders and furthermore, inflammatory cells inside the dermal papillae (red arrow); (**d**) dermis with elastic and collagen fibers with the appearance of a blurred reticulated meshwork (purple arrow).

At RCM examination, in healthy skin (see Figure 2), the granular and spinous layers display a regular honeycomb pattern (Figure 2b). The basal layer is characterized by pigmented skin cells arranged in a cobblestone pattern (bright structures). At the dermo-epidermal junction, basal keratinocytes and melanocytes are arranged in a circular pattern (bright rings) surrounding the dermal papillae (dark structures) with small blood vessels visible inside them (Figure 2c). Going deeper, elastic and collagen fibers are observed as bright fibrillar or reticular structures (Figure 2d). In atopic skin, due to the disrupted skin barrier, the granular and spinous layers show irregular cells with increased intercellular inflammatory infiltrate (inflammatory cells are visible as small bright structures) and spongiosis (large dark areas) (Figure 3b). Due to the thickening of the spinous layer in atopic skin, the basal layer, the dermal-epidermal junction (Figure 3c), and also the superficial part of the dermis may appear blurred. The papillary rings are irregular, the cellular borders almost disappear, and furthermore, inside the dermal papillae there are inflammatory cells (Figure 3c). The dermal elastic and collagen fibers can still be observed; however, their appearance resembles more of a blurred reticulated meshwork (Figure 3d). 

**Figure 4:**
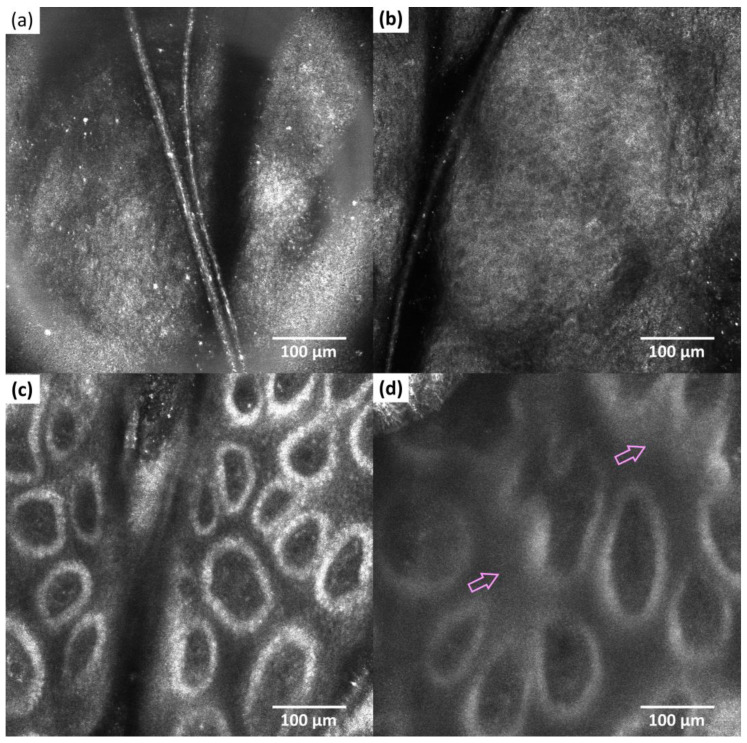
Reflectance confocal microscopy (RCM) images (500 × 500 μm) of the antecubital right fossae 3 weeks after treatment—right forearm (**a**) cornified layer; (**b**) granular/spinous layer with restored regular honeycomb pattern; (**c**) dermal-epidermal junction—visible bright rings surrounding the dermal papillae; decreased accumulation of inflammatory cells in the dermal papillae (**d**) fuzzy aspect of dermis with the appearance of a blurred reticulated meshwork (purple arrow).

Clinically, the evolution was favorable, the pruritus disappeared, and the lesions almost healed, leaving small erythematous-squamous plaques on the antecubital fossae. RCM examination was performed again after three weeks. The accumulation of inflammatory cells in the epidermis decreased (decreasing of the intercellular small bright structures), spongiosis was reduced (Figure 4b) and the papillary rings at the dermal-epidermal junction were reshaped (Figure 4c). However, the dermis maintained its fuzzy appearance resembling a blurred reticulated meshwork (Figure 4d).

## 3. Discussion

Atopic dermatitis is a common inflammatory skin disease with a major impact on the patient’s quality of life. When children and teenagers are affected, they can experience large discomfort by occurring symptoms, compared to adults [56,57]. In order to improve the quality of life of the patients, the treatment and the investigations should be chosen to have minimal impact during the treatment period and to obtain the best results. 

Topical calcineurin inhibitors are considered immunosuppressive agents because they suppress the synthesis of pro-inflammatory cytokines by interacting with the intercellular protein macrophilin-12 that can be found in LTh1 and LTh2. These molecules are involved in the development, persistence, and recurrence of skin lesions in AD [58]. The patient was treated with topical calcineurin inhibitors on the antecubital fossae because they are an alternative to the topical corticosteroids on the special sites (face, eyelid, neck, folds). These immunomodulating agents do not cause secondary atrophy, as dermatocorticoids do [47,55]. After the initial treatment, the lesions almost healed, but the maintenance treatment with topical calcineurin inhibitors or dermatocorticoids and emollients after shower is the key to success in preventing the recurrence of the lesions or the development of new ones.

RCM is useful in evaluating AD and can also be used to assess the response to the therapy [51,59]. It allows the direct investigation of exocytosis, spongiosis of the spinous and granular layer, parakeratosis, and the structural defects of the stratum corneum [51]. Depending on the difference between the refractive indices of the skin structures, the images are dark (hyporeflective surface) or bright (reflective surface), horizontal, and two-dimensional [51,52]. 

In our case, we were able to observe notable differences between the healthy skin, the atopic skin, and also the atopic cutaneous tissue after initial treatment. The main characteristics of atopic dermatitis were the interstitial inflammatory infiltrate, visualized as intercellular small bright structures in the epidermis and the spongiosis, visualized as dark areas compared with the surrounding epithelium in the granular and spinous layers. The regular aspects of the honeycomb pattern were also lost. For our patient, we were able to observe considerable improvements already after three weeks of treatment with decreased inflammatory infiltrate. Also, the bright rings surrounding the dermal papillae have reshaped. 

Furthermore, the reflectance confocal microscope allows the visualization of different areas of the lesion or different lesions at the same time, decreasing the total number of skin biopsies [51]. Unfortunately, it does not penetrate below the superficial dermis and also it can not differentiate precisely between the leukocyte subtypes in the inflammatory infiltrate [51,59,60,61].

## 4. Conclusions

RCM is a great tool for diagnosing and monitoring atopic skin lesions that currently require a skin biopsy. It can be used to observe the dynamic changes in the skin during treatment, such as the resolution of the inflammatory infiltrate in the epidermis and the reshaping of the papillary rings in the dermal-epidermal junction. In the subclinical stages of the inflammatory process, clinically, the skin appears healthy, but with RCM, the inflammatory infiltrate can be identified. Future developments, which will lead to the definition and validation of RCM criteria for the subclinical and clinical stages of AD, could help to improve the treatment and prevention strategies of the disease. Moreover, so far, proactive treatment is one of the best choices in order to prevent the relapse of the lesions or the development of new ones. 

## Data Availability

Not applicable.
